# Development and validation of an interpretable machine learning model for predicting central lymph node metastasis in papillary thyroid cancer

**DOI:** 10.3389/fonc.2026.1839870

**Published:** 2026-06-12

**Authors:** Li Zhou, Wei-ping Lu, Heng-lu Zhang, Min Wang, Hong-man Zhang, Di Yao, Song-qing Zhao

**Affiliations:** 1 Department of Endocrinology and Metabolism, The Affiliated Huaian No.1 People’s Hospital of Nanjing Medical University, Huaian, Jiangsu, China; 2 Department of Geriatrics, The Affiliated Huaian No.1 People’s Hospital of Nanjing Medical University, Huaian, Jiangsu, China

**Keywords:** blood-derived inflammatory markers, central lymph node metastasis, machine learning, papillary thyroid carcinoma, SHAP analysis

## Abstract

**Objective:**

To develop and validate a machine learning-based prediction model for central lymph node metastasis (CLNM) in papillary thyroid carcinoma (PTC) patients using routine blood test-derived inflammatory markers and clinical features.

**Methods:**

This retrospective study included 1,697 PTC patients. The cohort was randomly divided into training (70%) and validation (30%) sets. Clinical variables and inflammatory markers derived from routine blood tests including neutrophil-to-lymphocyte ratio, platelet-to-lymphocyte ratio, lymphocyte-to-monocyte ratio (LMR), and systemic immune-inflammation index (SII) were collected. LASSO regression was applied for feature selection, followed by development of eight machine learning algorithms. The optimal model was selected based on discrimination, calibration, and clinical utility. SHAP analysis was performed to enhance interpretability.

**Results:**

CLNM occurred in 516 patients (30.4%). LASSO regression identified 29 predictive features. The Stacking Ensemble model achieved superior performance with AUC of 0.988 in training and 0.923 in validation sets, significantly outperforming traditional logistic regression (AUC: 0.721). SHAP analysis revealed maximum tumor size as the most important predictor, followed by LMR, age, VEGF, and SII. Decision curve analysis demonstrated substantial clinical benefit across threshold probabilities.

**Conclusion:**

The machine learning-based model incorporating routine blood test-derived inflammatory markers and clinical features demonstrates excellent performance for CLNM prediction in PTC patients, providing a valuable tool for surgical decision-making and precision medicine approaches.

## Introduction

Papillary thyroid carcinoma (PTC) represents the most prevalent form of thyroid malignancy, accounting for approximately 85-90% of all thyroid cancer cases worldwide (1). Over the past three decades, the global incidence of thyroid cancer has risen markedly, with PTC accounting for the most pronounced increase (2). Despite the generally excellent prognosis associated with PTC, with 10-year survival rates exceeding 90%, central lymph node metastasis (CLNM) remains a significant clinical concern, occurring in 20-80% of patients and serving as a primary risk factor for disease recurrence and reoperation (3, 4).

The accurate preoperative prediction of CLNM is crucial for determining optimal surgical strategies and improving patient outcomes. Current imaging modalities, particularly ultrasonography, demonstrate limited sensitivity for detecting central lymph node metastasis, with reported sensitivity rates below 50% (5). This diagnostic limitation substantially complicates clinical decision-making. Furthermore, there remains significant controversy among different guidelines regarding central lymph node dissection: the 2015 American Thyroid Association guidelines suggest that prophylactic central compartment neck dissection may be considered for clinically node-negative patients, particularly for advanced primary tumors (T3 or T4), while some other guidelines recommend routine central lymph node dissection (6, 7). This ongoing controversy highlights the urgent need for reliable predictive models that can accurately identify patients at high risk for CLNM before surgical intervention.

Recent advances in machine learning have shown remarkable promise in medical prediction tasks, offering sophisticated analytical capabilities that surpass traditional statistical methods (8). A recent meta-analysis of 35 studies on AI-based prediction of thyroid cancer metastasis reported a pooled AUC of 0.82 and a pooled DOR of 9.45, supporting their diagnostic value while indicating room for improvement (9). The Least Absolute Shrinkage and Selection Operator (LASSO) regression has emerged as a powerful feature selection technique, effectively identifying the most relevant predictors while preventing overfitting. Additionally, the integration of interpretable artificial intelligence methods, such as SHapley Additive exPlanations (SHAP), has addressed the “black box” nature of complex machine learning models, enabling clinicians to understand the decision-making process (10, 11).

Inflammatory markers derived from routine blood tests have gained increasing attention as potential biomarkers for cancer progression and metastasis. The neutrophil-to-lymphocyte ratio (NLR), platelet-to-lymphocyte ratio (PLR), lymphocyte-to-monocyte ratio (LMR), and systemic immune-inflammation index (SII) reflect the complex interplay between systemic inflammation and immune response in cancer development. Several studies have reported significant correlations between elevated inflammatory markers and increased risk of lymph node metastasis in thyroid cancer, suggesting their potential utility as non-invasive predictive tools (12, 13).

However, comprehensive predictive models that integrate multiple inflammatory markers with clinical features using advanced machine learning techniques remain limited. Therefore, this study aimed to develop and validate a machine learning-based prediction model for CLNM in PTC patients using LASSO regression for feature selection and machine learning algorithms. We employed SHapley Additive exPlanations (SHAP) analysis to enhance model interpretability and provide actionable insights for clinical decision-making.

## Methods

### Study design and participants

The study was approved by Ethics Committees of The Affiliated Huaian No.1 People’s Hospital of Nanjing Medical University, and informed consent was taken from all individual participants ethics approval number “KY-2024-077-01”. Our institution is a high-volume regional referral center for thyroid surgery, with an annual surgical volume of approximately 900–1000 PTC cases. Between May 2022 and July 2024, all consecutive patients with pathologically confirmed PTC who underwent thyroidectomy with prophylactic central compartment dissection were screened. After applying the inclusion and exclusion criteria, 1,697 patients were enrolled, corresponding to approximately 63 patients per month, consistent with the institutional surgical volume.

Inclusion criteria: ① Postoperative pathological diagnosis of PTC; ② Unilateral thyroid lobectomy with isthmusectomy or total thyroidectomy, along with prophylactic central compartment lymph node dissection; ③ Complete preoperative laboratory examinations, imaging data, and surgical pathology records; ④ Available preoperative peripheral blood samples within one week before surgery.

Exclusion criteria: ① Presence of other malignant tumors or history of neck radiation; ② Coexistence of hematological disorders; ③ Coexistence of active systemic autoimmune diseases (excluding Hashimoto’s thyroiditis, such as systemic lupus erythematosus, rheumatoid arthritis, or other conditions requiring immunosuppressive therapy); ④ Preoperative occurrence of acute or chronic inflammation or other diseases affecting blood routine examination results; ⑤ Previous thyroid surgery history; ⑥ Incomplete preoperative clinical and pathologic data; ⑦ Patients with severe comorbidities that could significantly affect inflammatory markers (e.g., active malignancies, severe organ failure, immunosuppressive therapy).

The cohort was randomly divided into training (70%) and validation (30%) sets using stratified random sampling based on CLNM status to ensure identical distribution of the outcome variable (30.4% CLNM prevalence) in both sets. This stratification approach prevents imbalanced outcome distribution that could bias model development and validation.

### Data collection and variables

Clinical and laboratory data were systematically collected from electronic medical records. Demographic characteristics included age and gender. Laboratory parameters encompassed complete blood count indices, inflammatory ratios (NLR, PLR, MLR, LMR, SII, Aggregate Index of Systemic Inflammation, Eosinophil-to-Lymphocyte Ratio), thyroid function tests (thyroid stimulating hormone, free triiodothyronine, free thyroxine), thyroid-related antibodies, tumor markers (thyroglobulin, CEA, VEGF), and other biochemical parameters (parathyroid hormone, 25-Hydroxyvitamin D, calcitonin). Clinical features included presence of Hashimoto’s thyroiditis, vitamin D deficiency status (defined as serum 25-hydroxyvitamin D <20 ng/mL measured by chemiluminescence immunoassay), tumor characteristics (maximum diameter measured on preoperative ultrasound imaging, number of tumors), and the primary outcome variable of CLNM. VEGF was measured using enzyme-linked immunosorbent assay (ELISA) with a reference range of 62–707 pg/mL. All VEGF measurements were performed using the same commercial ELISA kit by trained laboratory personnel following standardized institutional protocols to ensure measurement consistency. Maximum tumor diameter was independently measured on preoperative ultrasound by two senior radiologists (each with more than 10 years of thyroid ultrasound experience), blinded to the pathology results. The mean of the two measurements was used for analysis; in cases of disagreement exceeding 2 mm, a third senior radiologist adjudicated. Inter-observer agreement for tumor size measurement was excellent (intraclass correlation coefficient, ICC = 0.93, 95% CI: 0.91–0.95). Given that the 1 cm cut-off is critical for T-staging and surgical decision-making, this dual-reader protocol was applied to minimize measurement variability. All postoperative specimens were independently reviewed by two senior thyroid pathologists, each with more than 10 years of experience in thyroid pathology. Discordant cases were resolved by consensus involving a third senior pathologist. Inter-observer agreement was substantial for both PTC diagnosis (Cohen’s κ = 0.86) and CLNM identification (Cohen’s κ = 0.91).

### Feature selection using LASSO regression

To identify the most relevant predictors for CLNM, we applied LASSO regression to all collected variables. LASSO regression performs automatic feature selection by shrinking less informative coefficients toward zero via L1 regularization toward. The optimal regularization parameter (λ) was determined using 10-fold cross-validation, selecting λ_min that minimized cross-validation error. Variables with non-zero coefficients at the optimal λ were retained for subsequent machine learning model development.

### Machine learning model development

Eight machine learning algorithms were implemented using the selected features: Logistic Regression, Random Forest (RF), Gradient Boosting Machine (GBM), Support Vector Machine (SVM), Neural Network, Convolutional Neural Network (CNN), AdaBoost, and Stacking Ensemble. Because the input data were tabular, the CNN was implemented as a one-dimensional architecture: features were reshaped into a 1×n vector and passed through two 1D convolutional layers (kernel size = 3, ReLU activation) followed by fully connected layers. This design was intended to capture local interactions among adjacent ordered laboratory indices and was included as an exploratory benchmark rather than a primary candidate model. For each algorithm, hyperparameter optimization was performed using grid search with 5-fold cross-validation to identify optimal parameter combinations. The Stacking Ensemble architecture employed RF, GBM, SVM, and Neural Network as base learners, with Logistic Regression serving as the meta-learner. Base learner predictions were combined through weighted probability averaging, where meta-learner weights were optimized using cross-validated predictions from the training set.

### Subgroup analysis

To evaluate model performance across different patient populations, we conducted subgroup analyses stratified by: (1) tumor size (≤1 cm vs. >1 cm), (2) presence of Hashimoto’s thyroiditis (yes vs. no), and (3) age groups (≤45 years vs. >45 years). Model discrimination (AUC) was assessed within each subgroup to determine consistency of performance.

### Model evaluation and validation

Model performance was assessed using multiple metrics including area under the receiver operating characteristic curve (AUC), accuracy, sensitivity, specificity, positive predictive value (PPV), negative predictive value (NPV), and F1-score. Receiver operating characteristic (ROC) curves were generated to evaluate discriminative ability. Model calibration was assessed using calibration plots comparing predicted probabilities with observed outcomes. Decision curve analysis (DCA) was conducted to evaluate clinical utility by calculating net benefit across different threshold probabilities compared to “treat all” and “treat none” strategies.

### Model interpretability analysis

To enhance model transparency, SHAP analysis was performed on the optimal model. SHAP values quantify each feature’s contribution to individual predictions, providing both global feature importance rankings and local explanations. SHAP summary plots, class-specific importance analysis, and decision plots were generated to visualize feature impacts and understand model decision-making processes. Feature importance was ranked by mean absolute SHAP values across all predictions.

### Statistical analysis

Continuous variables were presented as median with interquartile range (IQR) for non-normally distributed data and mean ± standard deviation for normally distributed data. Categorical variables were expressed as frequencies and percentages. Differences between training and validation sets were assessed using Mann-Whitney U test for continuous variables and chi-square test for categorical variables. All analyses were performed using Python 3.8 with scikit-learn, XGBoost, and SHAP libraries. Univariate comparisons between CLNM and non-CLNM groups were conducted using Mann-Whitney U test for continuous variables and chi-square test for categorical variables. Spearman correlation analysis was performed to assess multicollinearity among features, with correlation coefficients >0.8 considered highly correlated. Highly correlated features were examined, and one feature from each correlated pair was retained based on clinical relevance and predictive importance. Statistical significance was set at *P* < 0.05.

## Results

### Demographics and clinical characteristics of PTC patients

A total of 1,697 patients with pathologically confirmed PTC were included in this study. The cohort was randomly divided into a training set (n=1,187, 70%) and a validation set (n=510, 30%). The baseline characteristics are summarized in **Table 1**.

**Table 1:** Baseline characteristics of papillary thyroid carcinoma patients in training and validation sets.

Variable	Total (N = 1697)	Training set (N = 1187)	Validation set (N = 510)	Z/t/χ²	*P* value
Age (years)	47 (36-55)	47 (36-55)	47.5 (36.0-55.0)	0.151	0.880
WBC (×10⁹/L)	5.4 (4.5-6.4)	5.4 (4.5-6.4)	5.3 (4.5-6.3)	0.684	0.494
RBC (×10¹²/L)	4.5 (4.2-4.8)	4.5 (4.2-4.9)	4.5 (4.2-4.8)	0.930	0.352
Hemoglobin (g/L)	134 (125-144)	134 (125-144)	133 (124-143)	1.594	0.111
Hematocrit (%)	40.6 (38.1-43.5)	40.6 (38.2-43.6)	40.6 (37.7-43.2)	1.078	0.281
MCV (fL)	90.1 (87.4-92.8)	90.0 (87.3-92.8)	90.4 (87.6-92.7)	-0.778	0.437
MCH (pg)	29.8 (28.7-30.8)	29.8 (28.7-30.8)	29.8 (28.7-30.8)	0.045	0.964
MCHC (g/L)	329 (321-337)	329 (321-337)	329.0 (321.0-336.8)	1.029	0.303
Platelet count (×10⁹/L)	225 (186-266)	225 (186-266)	221.0 (186.0-268.8)	0.159	0.874
RDW-SD (fL)	41.6 (39.8-43.7)	41.6 (39.8-43.7)	41.5 (39.9-43.7)	0.394	0.693
RDW-CV (%)	12.6 (12.2-13.2)	12.7 (12.2-13.2)	12.6 (12.1-13.2)	1.337	0.181
PDW (fL)	12.4 (11.0-14.1)	12.4 (11.0-14.2)	12.4 (11.0-13.9)	0.885	0.376
MPV (fL)	10.6 (10.0-11.4)	10.6 (10.0-11.4)	10.6 (9.9-11.3)	1.304	0.192
PCT (%)	0.2 (0.2-0.3)	0.2 (0.2-0.3)	0.2 (0.2-0.3)	0.444	0.656
P-LCR (%)	29.9 (24.6-36.1)	29.9 (24.8-36.2)	29.9 (24.3-35.4)	1.188	0.235
Neutrophil count (×10⁹/L)	3.1 (2.5-3.9)	3.1 (2.5-4.0)	3.0 (2.5-3.7)	1.232	0.218
Lymphocyte count (×10⁹/L)	1.7 (1.4-2.1)	1.7 (1.4-2.1)	1.7 (1.4-2.1)	-0.260	0.795
Monocyte count (×10⁹/L)	0.3 (0.3-0.4)	0.3 (0.3-0.4)	0.3 (0.3-0.4)	-0.676	0.499
Neutrophil percentage (%)	58.6 ± 8.6	58.7 ± 8.5	58.3 ± 8.6	0.742	0.458
Lymphocyte percentage (%)	32.5 ± 7.7	32.4 ± 7.7	32.7 ± 7.8	-0.717	0.473
Monocyte percentage (%)	6.2 (5.3-7.3)	6.2 (5.3-7.3)	6.3 (5.3-7.5)	-1.160	0.246
Eosinophil percentage (%)	1.6 (1.0-2.5)	1.6 (1.0-2.5)	1.5 (1.0-2.5)	0.421	0.674
Basophil percentage (%)	0.5 (0.4-0.7)	0.5 (0.4-0.7)	0.5 (0.4-0.7)	0.324	0.744
TSH (mIU/L)	2.0 (1.4-3.0)	2.0 (1.4-3.0)	2.0 (1.4-2.9)	-0.289	0.772
FT3 (pmol/L)	5.0 (4.6-5.7)	5.0 (4.5-5.7)	5.1 (4.6-5.9)	-1.638	0.101
FT4 (pmol/L)	15.9 (13.7-17.5)	16.0 (13.9-17.6)	15.7 (13.4-17.4)	1.996	0.046
Anti-TPO (IU/mL)	12.5 (10.6-15.2)	12.5 (10.6-15.3)	12.5 (10.6-14.9)	0.513	0.604
Anti-TG (IU/mL)	16.6 (14.6-23.2)	16.6 (14.7-24.1)	16.5 (14.4-22.1)	1.300	0.194
Tg (ng/mL)	14.4 (8.8-26.7)	14.4 (8.7-27.0)	14.4 (9.0-25.3)	0.706	0.480
Calcitonin (pg/mL)	0.8 (0.5-3.0)	0.7 (0.5-2.7)	0.8 (0.5-4.0)	-1.165	0.226
PTH (pg/mL)	37.6 (27.8-47.5)	37.6 (28.2-47.0)	37.6 (27.1-49.6)	-0.033	0.974
25(OH)D (ng/mL)	15.3 (11.8-20.3)	15.3 (11.8-20.5)	15.1 (11.8-19.6)	0.779	0.436
CEA (ng/mL)	1.4 (0.9-1.9)	1.4 (0.9-1.9)	1.4 (0.9-1.9)	0.337	0.736
VEGF (pg/mL)	245.6 (158.3–412.7)	246.1 (157.9–415.2)	244.3 (159.1–408.5)	0.412	0.680
Maximum tumor size (cm)	0.8 (0.5-1.2)	0.8 (0.5-1.2)	0.7 (0.5-1.2)	1.079	0.279
NLR	1.8 (1.4-2.3)	1.8 (1.4-2.4)	1.8 (1.4-2.2)	1.176	0.240
PLR	131.2 (105.6-165.9)	130.7 (106.2-165.9)	132.7 (102.9-165.6)	0.164	0.870
MLR	0.2 (0.2-0.2)	0.2 (0.2-0.2)	0.2 (0.2-0.2)	-0.160	0.873
SII	406.2 (297.1-558.9)	408.1 (297.3-564.5)	397.8 (296.6-537.7)	0.815	0.415
AISI	0.6 (0.4-0.9)	0.6 (0.4-0.9)	0.6 (0.4-0.8)	0.883	0.377
LMR	5.1 (4.1-6.3)	5.1 (4.1-6.4)	5.1 (4.1-6.3)	0.160	0.873
ELR	0.0 (0.0-0.1)	0.0 (0.0-0.1)	0.0 (0.0-0.1)	0.728	0.467
Sex					
Male	370 (21.8)	264 (22.2)	106 (20.8)	0.363	0.547
Female	1327 (78.2)	923 (77.8)	404 (79.2)		
Hashimoto’s thyroiditis					
No	1338 (78.8)	933 (78.6)	405 (79.4)	0.096	0.757
Yes	359 (21.2)	254 (21.4)	105 (20.6)		
Vitamin D deficiency					
No	438 (25.8)	317 (26.7)	121 (23.7)	1.503	0.220
Yes	1259 (74.2)	870 (73.3)	389 (76.3)		
Tumor number					
Single	1224 (72.1)	860 (72.5)	364 (71.4)	0.156	0.692
Multiple	473 (27.9)	327 (27.5)	146 (28.6)		
CLNM					
No	1181 (69.6)	826 (69.6)	355 (69.6)	0.000	1.000
Yes	516 (30.4)	361 (30.4)	155 (30.4)		

Continuous variables are presented as median (interquartile range) for non-normally distributed data and mean ± standard deviation for normally distributed data. Categorical variables are presented as frequency (percentage).

WBC, white blood cell count; RBC, red blood cell count; MCV, mean corpuscular volume; MCH, mean corpuscular hemoglobin; MCHC, mean corpuscular hemoglobin concentration; RDW-SD, red cell distribution width-standard deviation; RDW-CV, red cell distribution width-coefficient of variation; PDW, platelet distribution width; MPV, mean platelet volume; PCT, plateletcrit; P-LCR, platelet large cell ratio; TSH, thyroid stimulating hormone; FT3, free triiodothyronine; FT4, free thyroxine; Anti-TPO, anti-thyroid peroxidase antibody; Anti-TG, anti-thyroglobulin antibody; Tg, thyroglobulin; PTH, parathyroid hormone; 25(OH)D, 25-hydroxyvitamin D; CEA, carcinoembryonic antigen; VEGF, vascular endothelial growth factor; NLR, neutrophil-to-lymphocyte ratio; PLR, platelet-to-lymphocyte ratio; MLR, monocyte-to-lymphocyte ratio; SII, systemic immune-inflammation index; AISI, aggregate index of systemic inflammation; LMR, lymphocyte-to-monocyte ratio; ELR, eosinophil-to-lymphocyte ratio; CLNM, central lymph node metastasis.

The median age was 47 years (IQR: 36-55), with female predominance (78.2%). CLNM occurred in 516 patients (30.4%), with identical distribution in both training and validation sets. Key inflammatory markers included NLR (median: 1.8, IQR: 1.4-2.3), PLR (median: 131.2, IQR: 105.6-165.9), and SII (median: 406.2, IQR: 297.1-558.9). VEGF levels showed a wide distribution across the cohort (median: 245.6 pg/mL, IQR: 158.3–412.7), with comparable distributions between training and validation sets (P = 0.680). The median tumor size was 0.8 cm (IQR: 0.5-1.2), with single tumors in 72.1% of patients.

Hashimoto’s thyroiditis was present in 21.2% of patients, while vitamin D deficiency occurred in 74.2%. No statistically significant differences were observed between training and validation sets for any baseline characteristics (all *P* > 0.05), confirming successful randomization.

### Comparison of clinical characteristics between CLNM and non-CLNM groups

Significant differences were observed between patients with and without CLNM (**Supplementary Table 1**). Patients with CLNM were younger (median age: 43 vs. 48 years, *P* < 0.001), had larger tumors (median size: 1.1 vs. 0.7 cm, *P* < 0.001), and more frequently presented with multiple tumors (38.6% vs. 23.5%, *P* < 0.001). Inflammatory markers showed distinct patterns: CLNM patients exhibited higher SII (median: 458.3 vs. 385.2, *P* < 0.001), PLR (median: 142.5 vs. 126.8, P = 0.002), and lower LMR (median: 4.7 vs. 5.3, *P* < 0.001). VEGF levels were significantly elevated in the CLNM group (*P* = 0.015). Spearman correlation analysis revealed moderate correlations among inflammatory markers (SII-NLR: r=0.72, PLR-SII: r=0.65), but no strong multicollinearity (all r<0.8) that would necessitate feature removal.

### Feature selection by LASSO regression

**Figure 1:**
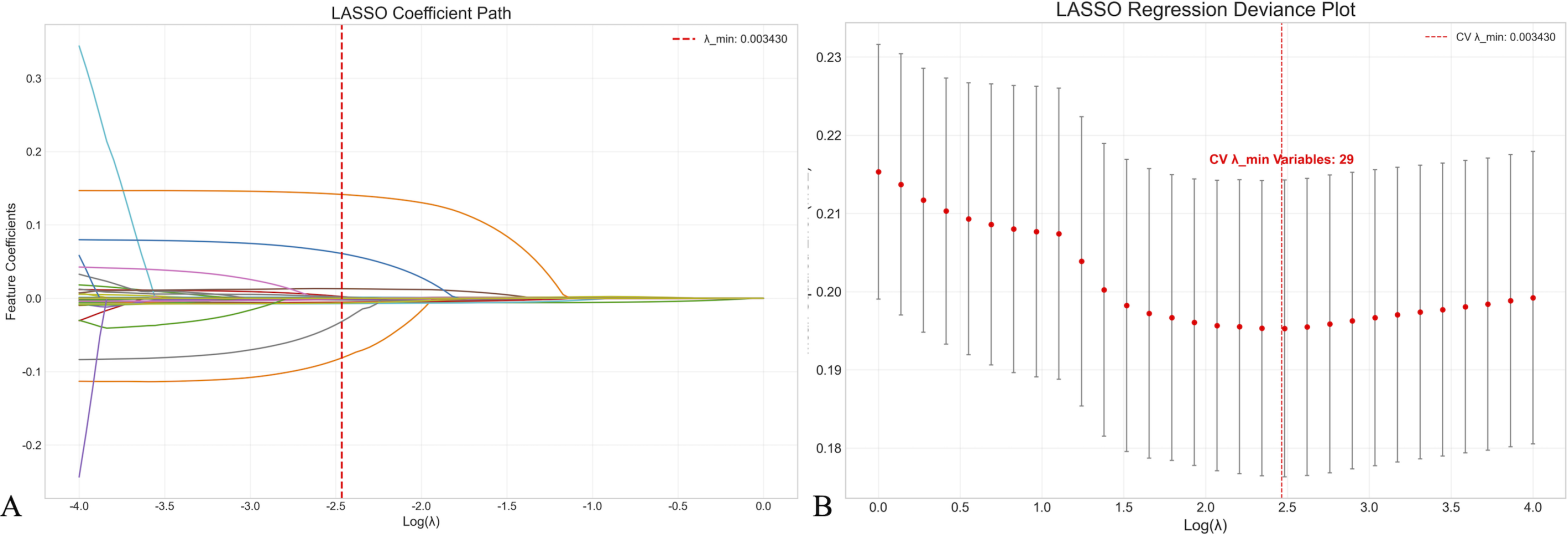
Feature selection using LASSO regression analysis. **(A)** LASSO coefficient path showing the shrinkage of regression coefficients as the regularization parameter (λ) increases. Each colored line represents a different variable, with coefficients gradually approaching zero as λ increases. The red dashed line indicates the optimal λ value (λ_min = 0.003430). **(B)** LASSO regression deviance plot with 10-fold cross-validation. The red dashed line marks the optimal λ_min value that minimizes cross-validation error, resulting in the selection of 29 variables for subsequent machine learning model development.

To identify the most relevant predictors for CLNM, we applied LASSO regression to all collected clinical variables. As illustrated in **Figures 1A, B**, LASSO regression analysis identified the optimal feature set for CLNM prediction.

**Figure 1A** shows the LASSO coefficient path, illustrating how the regression coefficients of different variables change as the regularization parameter (λ) increases. As λ increases, less important features are progressively shrunk to zero, effectively removing them from the model. **Figure 1B** presents the LASSO regression deviance plot with 10-fold cross-validation, where the optimal λ value (λ_min = 0.003430) was determined at the minimum cross-validation error.

At the optimal λ_min value, 29 variables were selected as the most predictive features for CLNM. Prior to LASSO selection, univariate analysis identified 35 variables with significant differences between CLNM and non-CLNM groups (*P* < 0.05, **Supplementary Table 1**). Correlation analysis confirmed no severe multicollinearity among selected features (maximum correlation coefficient: 0.72 between SII and NLR). These variables demonstrated non-zero coefficients and were retained for subsequent machine learning model development.

### Machine learning model development and performance comparison

**Figure 2:**
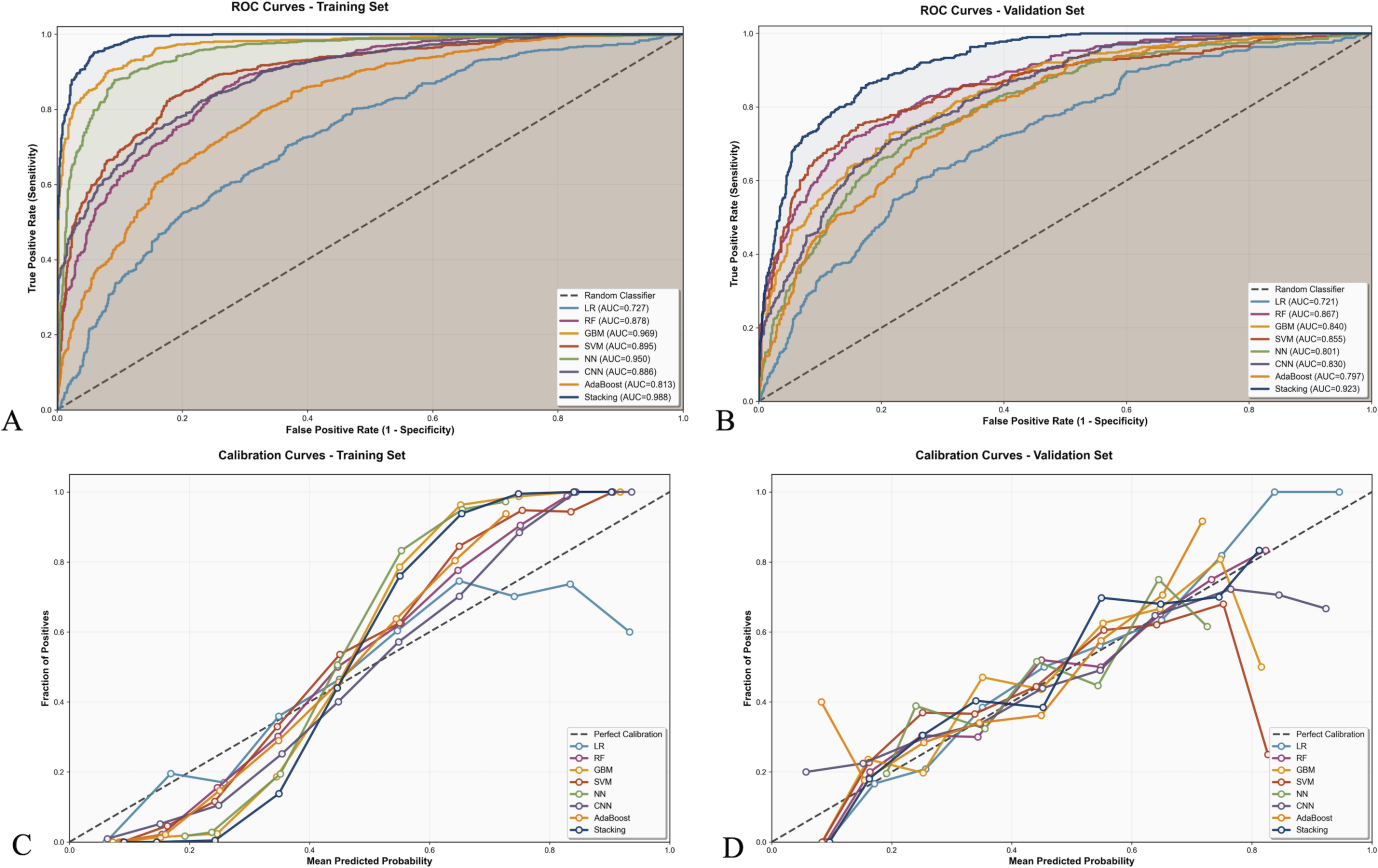
Performance evaluation of machine learning models for central lymph node metastasis prediction. **(A)** Receiver operating characteristic (ROC) curves for eight machine learning models in the training set. **(B)** ROC curves for the same models in the validation set. **(C)** Calibration curves for all models in the training set, comparing predicted probabilities with observed outcomes. **(D)** Calibration curves for all models in the validation set. The Stacking Ensemble model (dark blue) demonstrated superior performance with the highest AUC values in both training (0.988) and validation (0.923) sets. Perfect calibration is represented by the diagonal dashed line.

Using the 29 variables selected by LASSO regression, we developed eight machine learning algorithms for CLNM prediction. Performance metrics are presented in **Table 2**, with ROC and calibration curves shown in **Figure 2**.

**Table 2:** Performance metrics of machine learning models for central lymph node metastasis prediction.

Model	AUC	AUC 95% CI	Accuracy	Sensitivity	Specificity	PPV	NPV	F1 Score	Loss
Training Set									
LR	0.727	0.702-0.751	0.660	0.656	0.664	0.577	0.734	0.614	0.608
RF	0.878	0.862-0.895	0.785	0.845	0.742	0.696	0.873	0.764	0.471
GBM	0.969	0.961-0.976	0.834	0.980	0.731	0.718	0.982	0.829	0.368
SVM	0.895	0.879-0.909	0.775	0.914	0.677	0.664	0.919	0.770	0.453
Neural Network	0.950	0.938-0.960	0.885	0.902	0.873	0.832	0.927	0.866	0.437
CNN	0.886	0.871-0.903	0.777	0.850	0.727	0.685	0.874	0.758	0.446
AdaBoost	0.813	0.791-0.834	0.741	0.644	0.809	0.702	0.764	0.671	0.547
Stacking Ensemble	0.988	0.985-0.992	0.939	0.917	0.954	0.933	0.943	0.925	0.315
Validation Set									
LR	0.721	0.691-0.750	0.672	0.635	0.688	0.465	0.815	0.537	0.556
RF	0.867	0.846-0.885	0.736	0.850	0.688	0.537	0.915	0.658	0.476
GBM	0.840	0.817-0.860	0.701	0.842	0.640	0.500	0.905	0.628	0.507
SVM	0.855	0.831-0.879	0.726	0.850	0.673	0.526	0.913	0.650	0.504
Neural Network	0.801	0.775-0.826	0.714	0.751	0.698	0.515	0.868	0.611	0.529
CNN	0.830	0.808-0.853	0.740	0.749	0.736	0.548	0.873	0.633	0.474
AdaBoost	0.797	0.770-0.822	0.754	0.512	0.857	0.605	0.805	0.555	0.544
Stacking Ensemble	0.923	0.909-0.937	0.785	0.876	0.721	0.678	0.938	0.764	0.398

AUC, area under the curve; CI, confidence interval; PPV, positive predictive value; NPV, negative predictive value; LR, Logistic Regression; RF, Random Forest; GBM, Gradient Boosting; SVM, support vector machine; CNN, convolutional neural network.

In the training set, Stacking Ensemble achieved the highest AUC of 0.988 (95% CI: 0.985-0.992), followed by GBM (AUC: 0.969) and Neural Network (AUC: 0.950). In the validation set, Stacking Ensemble maintained superior performance with AUC of 0.923 (95% CI: 0.909-0.937), while RF showed robust performance (AUC: 0.867) and SVM achieved AUC of 0.855.

The Stacking Ensemble demonstrated minimal overfitting with the smallest performance gap between training and validation sets (ΔAUC = 0.065). All machine learning models significantly outperformed traditional logistic regression (validation AUC: 0.721). Calibration analysis revealed good agreement between predicted and observed probabilities for most models, with Stacking Ensemble showing optimal balance between discrimination and calibration.

### Clinical utility evaluation and model selection

**Figure 3:**
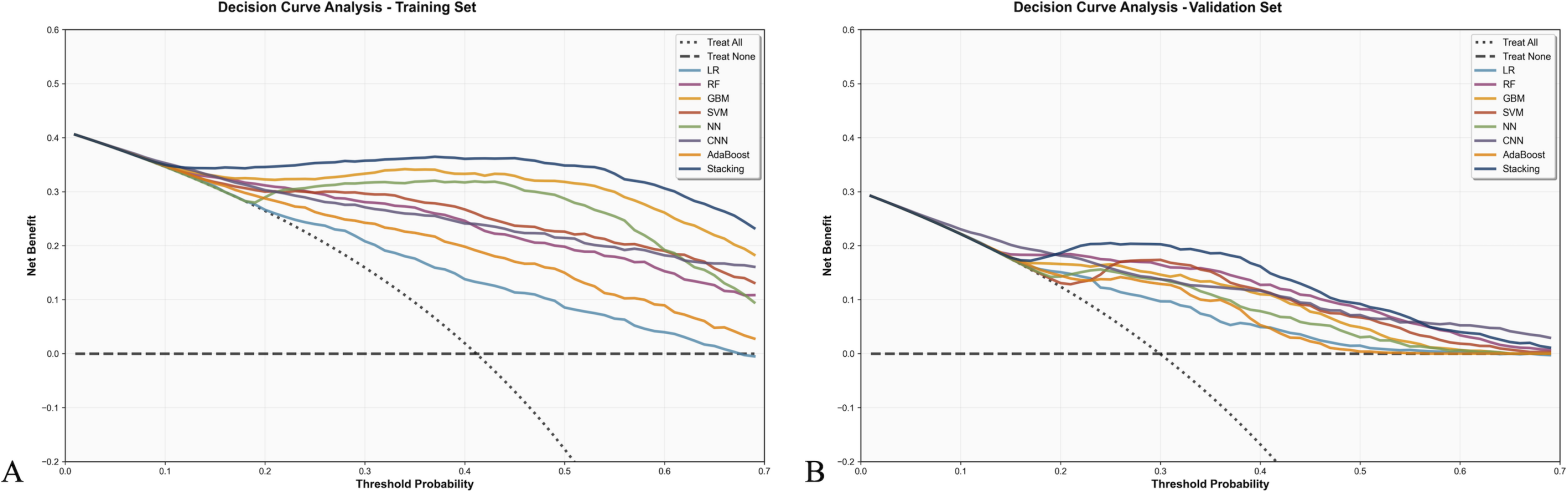
Decision curve analysis for clinical utility assessment. **(A)** Decision curve analysis in the training set showing net benefit across different threshold probabilities. **(B)** Decision curve analysis in the validation set. All machine learning models demonstrated positive net benefit compared to “treat all” (dotted line) and “treat none” (dashed line) strategies within the threshold probability range of 0.1 to 0.5. The Stacking Ensemble model (dark blue) consistently showed the highest net benefit across most threshold probabilities, indicating superior clinical utility for decision-making.

DCA was performed to evaluate the clinical utility of the developed models. Decision curve analysis (**Figures 3A, B**) confirmed that all machine learning models provided substantial net clinical benefit compared to “treat all” or “treat none” strategies across a wide range of threshold probabilities.

In both training and validation sets, the models showed positive net benefit within the threshold probability range of 0.1 to 0.6, indicating clinical utility for decision-making. The Stacking Ensemble consistently demonstrated the highest net benefit across most threshold probabilities. Based on the comprehensive evaluation of discrimination, calibration, and clinical utility, we selected the Stacking Ensemble as the optimal model for CLNM prediction. The model maintained similar performance between training and validation sets, confirming good generalizability and clinical applicability for CLNM prediction in PTC patients.

### Feature importance analysis of the optimal model

**Figure 4:**
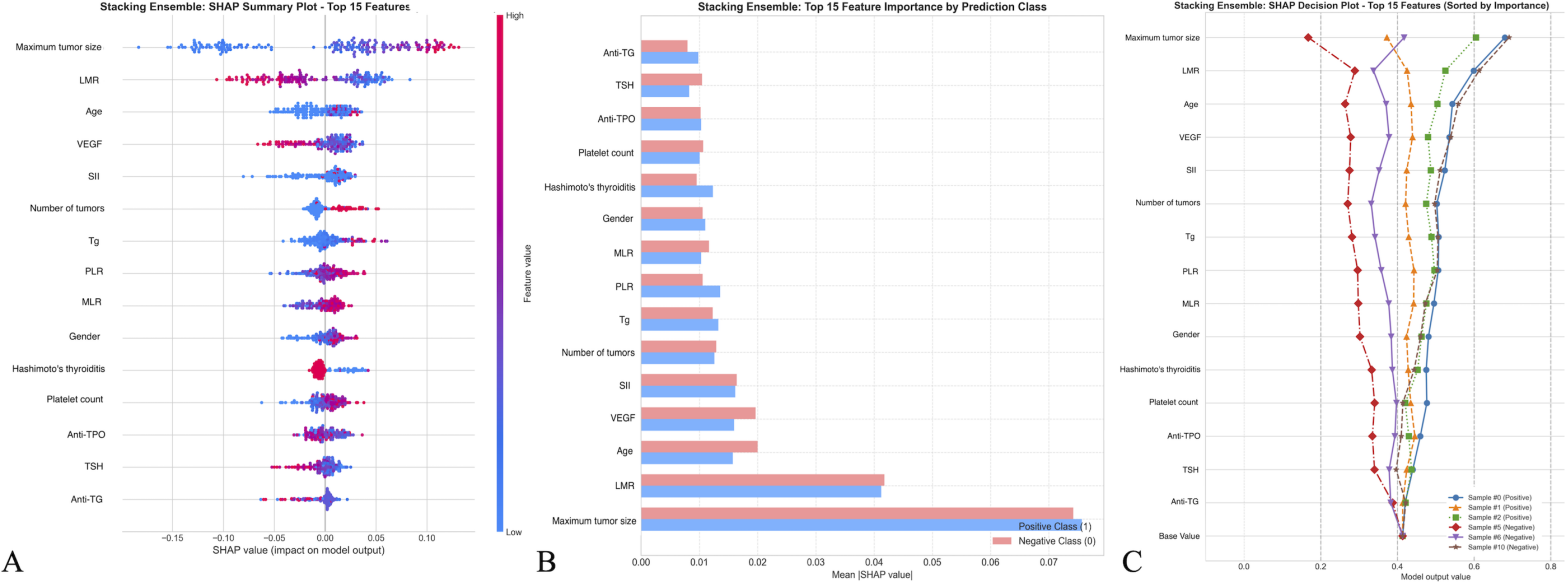
SHAP analysis for model interpretability and feature importance. **(A)** SHAP summary plot for the top 15 features showing the distribution of SHAP values and their impact on model predictions. Each dot represents a patient, with colors indicating feature values (red = high, blue = low). Features are ranked by mean absolute SHAP values. **(B)** Class-specific feature importance analysis comparing the mean absolute SHAP values for positive (CLNM) and negative (non-CLNM) prediction classes. **(C)** SHAP decision plot illustrating individual prediction pathways for representative patients, showing how each feature contributes to the final prediction from baseline probability. Different colored trajectories represent distinct patient profiles converging toward positive or negative CLNM outcomes.

To enhance model interpretability, we performed SHAP analysis on the selected Stacking Ensemble model to identify the most influential features for CLNM prediction (**Table 3**, **Figure 4**).

**Table 3:** Top 15 feature importance rankings based on SHAP analysis of the optimal stacking ensemble model.

Features	Mean |SHAP|	Std |SHAP|	Mean SHAP	Rank
Maximum tumor size	0.074713	0.039917	0.001620	1
LMR	0.041548	0.020799	-0.005268	2
Age	0.018418	0.011494	-0.004000	3
VEGF	0.018268	0.013380	0.001440	4
SII	0.016318	0.012800	0.001052	5
Number of tumors	0.012758	0.009263	-0.000440	6
Tg	0.012643	0.012136	0.002077	7
PLR	0.011661	0.010567	0.000653	8
MLR	0.011138	0.008370	-0.001707	9
Gender	0.010707	0.009083	0.000978	10
Hashimoto’s thyroiditis	0.010559	0.009114	0.000842	11
Platelet count	0.010419	0.008993	0.000368	12
Anti-TPO	0.010233	0.007069	0.000017	13
TSH	0.009631	0.009937	-0.002199	14
Anti-TG	0.008639	0.011216	-0.003651	15

SHAP, SHapley Additive exPlanations; LMR, Lymphocyte-to-Monocyte Ratio; VEGF, Vascular Endothelial Growth Factor; SII, Systemic Immune-Inflammation Index; Tg, Thyroglobulin; PLR, Platelet-to-Lymphocyte Ratio; Anti-TG, Anti-Thyroglobulin; Anti-TPO, Anti-Thyroid Peroxidase; MLR, Monocyte-to-Lymphocyte Ratio; TSH, Thyroid Stimulating Hormone.

Maximum tumor size emerged as the most important predictor with the highest mean |SHAP| value of 0.074713, followed by LMR (0.041548), age (0.018418), VEGF (0.018268), and SII (0.016318).

The SHAP summary plot (**Figure 4A**) revealed distinct patterns of feature influence: larger tumor sizes (red dots) consistently drove predictions toward higher CLNM probability (positive SHAP values), confirming its role as the primary risk factor. Higher LMR values (red dots) predominantly reduced CLNM risk (negative SHAP values), indicating its protective immunological effect. Interestingly, younger age (blue dots) was associated with increased CLNM risk, while elevated inflammatory markers including SII, PLR, and elevated VEGF levels generally increased metastasis probability. Multiple tumors were associated with increased CLNM risk, reflecting the aggressive nature of multifocal disease. The class-specific importance analysis (**Figure 4B**) demonstrated that maximum tumor size contributed most strongly to positive CLNM predictions, while LMR was the dominant protective factor. The SHAP decision plot (**Figure 4C**) illustrated individual prediction pathways, showing how each patient’s feature combination sequentially influenced the final prediction from baseline probability, with different colored trajectories representing distinct patient profiles converging toward positive or negative CLNM outcomes.

### Subgroup analysis results

The Stacking Ensemble model demonstrated consistent performance across different subgroups (**Supplementary Table 2**). For tumor size stratification, the model achieved AUC of 0.901 (95% CI: 0.878-0.924) for tumors ≤1 cm and 0.935 (95% CI: 0.915-0.955) for tumors >1 cm. In patients with Hashimoto’s thyroiditis, the AUC was 0.918 (95% CI: 0.892-0.944), compared to 0.925 (95% CI: 0.908-0.942) in patients without thyroiditis. Age-stratified analysis showed AUC of 0.931 (95% CI: 0.912-0.950) for patients ≤45 years and 0.916 (95% CI: 0.895-0.937) for patients >45 years. These results confirm robust model generalizability across clinically relevant patient subgroups.

### SHAP value distribution analysis

**Figure 5:**
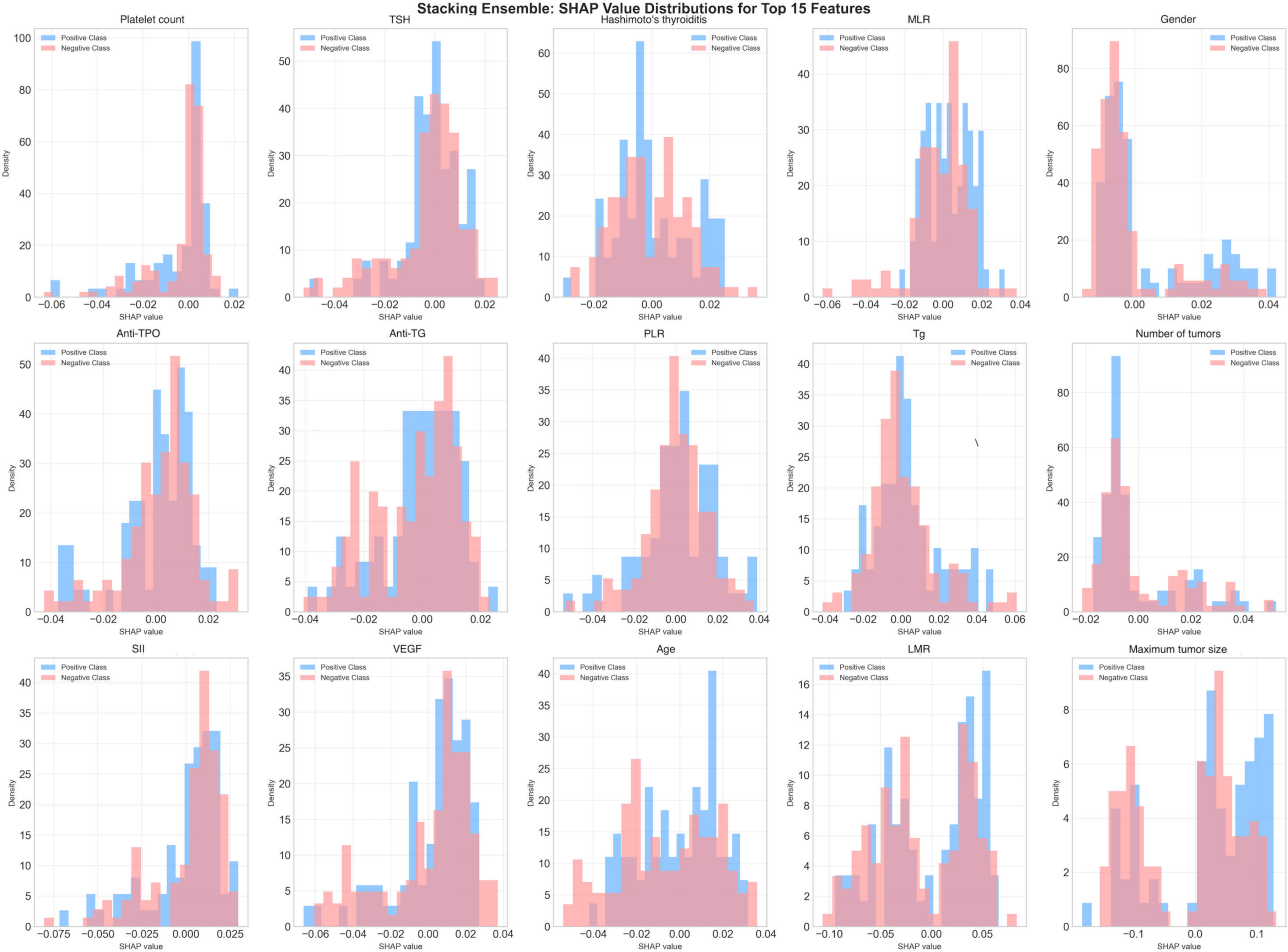
SHAP value distributions for top 15 predictive features stratified by prediction class. Distribution of SHAP values for the top 15 features stratified by prediction class (positive CLNM in blue, negative CLNM in red). Each subplot shows the density distribution of SHAP values for a specific feature, demonstrating how feature contributions differ between patients with and without central lymph node metastasis. Clear separation between classes indicates the discriminative power of each feature. Maximum tumor size, LMR, and age show the most distinct distribution patterns between positive and negative cases.

To examine feature contributions across different prediction classes, we analyzed SHAP value distributions for the top 15 features (**Figure 5**). Maximum tumor size showed rightward-shifted distributions in positive CLNM cases, confirming larger tumors increase metastasis risk. LMR demonstrated opposite patterns with negative SHAP values in negative cases, reinforcing its protective role. Age displayed bimodal distribution with younger patients more likely to have positive CLNM outcomes.

Inflammatory markers (SII, PLR, VEGF) showed rightward shifts for positive cases, indicating elevated inflammatory states increase CLNM risk. The clear separation of SHAP distributions between classes for key features validates the model’s ability to capture clinically meaningful patterns.

## Discussion

This study developed a comprehensive machine learning-based prediction model for CLNM in PTC patients, demonstrating superior performance compared to traditional approaches through advanced feature selection, ensemble learning, and interpretability analysis.

The Stacking Ensemble model achieved a strong validation-set AUC of 0.923, substantially outperforming conventional logistic regression (AUC: 0.721). The minimal overfitting (ΔAUC = 0.065) indicates robust generalizability crucial for clinical implementation. This superior performance aligns with recent findings by Lixandru-Petre et al (14), who highlighted the transformative potential of machine learning in thyroid cancer detection and metastasis prediction. The success demonstrates that sophisticated algorithmic approaches can capture complex non-linear relationships that traditional statistical methods cannot detect (15).

Our model’s performance compares favorably with recent studies in thyroid cancer prediction. While Feng et al (1) developed a radiomics-driven nomogram achieving good performance for specific anatomical regions, and Wang et al. demonstrated AI value using CT imaging (16), our comprehensive approach offers distinct advantages. By incorporating clinical variables, laboratory parameters, and inflammatory markers, our model utilizes readily available, cost-effective parameters suitable for widespread implementation, particularly in resource-limited settings.

The substantial improvement over Liu et al (2), who achieved moderate performance using traditional LASSO regression alone, stems from our sophisticated ensemble learning methodology combining predictions from multiple diverse base learners through a meta-learner architecture.

Our validation AUC of 0.923 compares favorably with previously reported prediction models for CLNM in PTC. Gao et al. l (2) constructed a nomogram with an AUC of approximately 0.737 using traditional logistic regression on clinical variables; Jiang et al. (3) reported a risk-scoring model with an AUC of 0.806; Chen et al. (4) developed a cervical lymph node prediction model reaching an AUC of 0.812; ultrasound-video-based deep learning by Zhang et al. (5) achieved an AUC of 0.89; and the CT-based AI model of Wang et al. (15) achieved an AUC of 0.86. The improvement of our approach stems from a sophisticated ensemble learning methodology combining predictions from multiple diverse base learners through a meta-learner architecture, allowing it to capture aspects of the data that single algorithms might miss. This stacking approach captures different aspects of underlying data patterns that single algorithms might miss. The integration of multiple algorithms including Logistic Regression, RF, GBM, SVM, Neural Network, CNN, and AdaBoost ensures robust performance across different data patterns while reducing overfitting risk.

Furthermore, our subgroup analyses demonstrated that the Stacking Ensemble maintained robust performance across different patient populations, including those stratified by tumor size, Hashimoto’s thyroiditis status, and age groups. This consistency validates the model’s applicability across diverse clinical scenarios. The superior performance of Stacking Ensemble over individual algorithms can be attributed to its ability to leverage complementary strengths of different base learners: RF captures non-linear interactions, GBM handles complex sequential patterns, SVM excels in high-dimensional spaces, and Neural Networks model intricate feature relationships. The meta-learner then optimally combines these diverse perspectives, reducing the risk of overfitting inherent in any single algorithm while maximizing predictive accuracy.

The AUC for tumors ≤1 cm (0.901, 95% CI: 0.878–0.924) was modestly lower than that for tumors >1 cm (0.935, 95% CI: 0.915–0.955), with overlapping 95% confidence intervals indicating that the difference is unlikely to be clinically meaningful. Nevertheless, because PTC microcarcinomas represent a large and growing proportion of clinical practice—and constitute the population in which the decision to perform prophylactic central neck dissection is most contested—even a modest reduction in discrimination warrants attention. Further refinement, potentially through incorporation of ultrasound radiomic features or molecular markers such as BRAF V600E, may be required to optimize model performance specifically in this subgroup.

The interpretability challenge inherent in complex machine learning models has been addressed through our integration of SHAP analysis, providing both global feature importance rankings and individual patient-level explanations, effectively addressing the “black box” limitation. As emphasized by Ponce-Bobadilla et al (17), SHAP’s value in building clinician trust cannot be overstated.

Through our feature analysis, we revealed the critical importance of inflammatory markers including the SII, LMR, PLR, and MLR in predicting CLNM. These findings align with growing evidence supporting the role of systemic inflammation in cancer progression and metastasis. Chen et al (12) demonstrated that SII-based models effectively predict central lymph node metastasis in clinically nodal-negative PTC patients, establishing SII as an independent risk factor. Our SHAP analysis revealed that elevated SII and PLR values increased metastasis probability, while higher LMR and MLR values provided protective effects, reflecting the complex interplay between immune surveillance and tumor progression.

The biological mechanisms underlying these associations involve coordinated actions of multiple cell types within the tumor-immune microenvironment. Elevated SII reflects neutrophils acquiring tumor-promoting phenotypes by secreting matrix metalloproteinases, reactive oxygen species, and pro-angiogenic factors that facilitate tumor invasion (13). Neutrophil heterogeneity, particularly immature subsets expressing higher levels of PD-L1, significantly influences T-cell immunity and tumor aggressiveness in thyroid cancer (18). Increased PLR reflects multiple platelet-mediated mechanisms promoting cancer metastasis, including formation of tumor cell-platelet aggregates that protect circulating tumor cells and release of platelet-derived growth factors (19).

Conversely, LMR and MLR serve as protective factors reflecting the critical roles of monocytes and lymphocytes in anti-tumor immunity. The process of monocyte differentiation into tumor-associated macrophages is regulated by CCR2 signaling, and its abnormal activation promotes epithelial-mesenchymal transition through secretion of TGF-β, VEGF, and IL-10 (20). Lymphocytes, particularly CD8+ T cells and natural killer cells, exert anti-tumor effects through direct cytotoxicity and immune surveillance mechanisms (21). VEGF, which has significant predictive value in our model, plays a key regulatory role by directly promoting tumor angiogenesis and reshaping the immune microenvironment through inhibiting dendritic cell maturation and promoting regulatory T cell expansion.

Our comprehensive univariate analysis and correlation assessment strengthen the robustness of feature selection. By demonstrating significant differences in 35 variables between CLNM and non-CLNM groups before LASSO regression, we established a clinically meaningful feature pool. The absence of severe multicollinearity (r<0.8) ensures that our model captures independent biological signals rather than redundant information, enhancing both interpretability and predictive efficiency. This rigorous feature screening process, combining statistical significance testing, correlation analysis, and LASSO regularization, represents a methodologically sound approach to biomarker selection that balances clinical relevance with model parsimony.

Our analysis revealed that younger age was associated with increased CLNM risk, consistent with Jiang et al (3). Although traditional risk stratification systems generally favor younger PTC patients in terms of overall survival, long-term survival and locoregional metastatic propensity are biologically distinct phenomena. Several converging mechanisms explain this pattern: (1) a higher prevalence of aggressive molecular alterations, particularly BRAF V600E and TERT promoter mutations, in younger PTC patients, which drive lymph node metastasis and extrathyroidal extension (22); (2) epigenetic dysregulation, including aberrant DNA methylation of tumor-suppressor genes and altered microRNA expression, promoting epithelial–mesenchymal transition and lymphatic dissemination; (3) a more active tumor immune-inflammatory microenvironment in younger patients, characterized by enhanced neutrophil extracellular trap formation, increased matrix metalloproteinase expression, and VEGF-mediated lymphangiogenesis, which facilitates lymph node colonization (23); and (4) age-related differences in thyroid lymphatic drainage and vascular density that may further influence metastatic routes (24). Together, these factors underscore the importance of age-stratified risk assessment and may justify more aggressive surgical strategies in younger PTC patients despite their favorable long-term prognosis (25). Additionally, the identification of multifocal tumors as a significant CLNM risk factor supports the concept that tumor biology, rather than size alone, plays a crucial role in determining metastatic potential (26).

The robust performance of our model suggests excellent potential for clinical implementation. The ongoing controversy surrounding prophylactic central neck dissection in clinically node-negative PTC patients underscores the critical need for accurate preoperative risk assessment tools (27). Our model provides objective, quantitative risk estimates that can inform shared decision-making between surgeons and patients. In clinical practice, this predictive tool could be integrated into routine preoperative workflows, utilizing easily available laboratory parameters to stratify patients into high-risk and low-risk categories for CLNM (28). High-risk patients would benefit from prophylactic central compartment dissection, potentially preventing future reoperation, while low-risk patients could safely undergo thyroidectomy alone, avoiding unnecessary surgical complications. This represents a paradigm shift toward precision medicine in thyroid cancer management (29).

While our comprehensive model achieved excellent performance using 29 features including VEGF, we acknowledge that certain biomarkers like VEGF are not routinely measured in all clinical settings, which may limit the model’s immediate widespread applicability. To address this practical concern, we conducted a sensitivity analysis excluding VEGF and other non-routine markers, developing a simplified model using only commonly available clinical and laboratory parameters. The simplified model included eight features: age, gender, maximum tumor size, number of tumors, NLR, PLR, LMR, and SII. Using these eight features only, the model achieved an AUC of 0.896 (95% CI: 0.879–0.913) in the validation set, representing only a modest decrease from the full 29-feature model (AUC 0.923). This finding suggests that our approach can be adapted for resource-limited settings while maintaining clinically acceptable performance. Future implementation could offer both a comprehensive version for advanced centers with complete biomarker panels and a simplified version for broader clinical use.

Several imaging modalities have been explored for preoperative CLNM prediction in PTC. Ultrasound remains the first-line modality given its low cost, wide accessibility, lack of ionizing radiation, and ease of bedside performance; Zhang et al. l (5) achieved an AUC of 0.89 using ultrasound-video-based deep learning, though conventional US is operator-dependent and shows limited sensitivity (<50%) for CLNM detection. CT-based AI offers better visualization of deep cervical structures (Wang et al (16) AUC 0.86), but entails ionizing radiation, contrast use, and higher cost. Our blood-based approach is complementary rather than competing—providing universally available, low-cost, objective, and operator-independent inputs derived from routine preoperative tests. As Feng et al (1) demonstrated, combined clinical-laboratory-imaging models outperform single modalities; future work should therefore integrate our inflammatory marker model with ultrasound features, leveraging US’s accessibility together with the objectivity of laboratory markers to build a pragmatic multimodal framework suitable for both well-resourced and resource-limited settings. Regarding clinical translation and implementation, the integration of our model into routine clinical workflows represents a critical next step. To facilitate widespread adoption, we propose developing an online risk calculator or mobile application that allows clinicians to input readily available patient parameters and obtain real-time CLNM risk predictions with individualized probability scores. Such digital tools have demonstrated success in other oncological contexts by providing point-of-care decision support.

With 516 CLNM events and 29 retained predictors, our events-per-predictor ratio was approximately 17.8, within the commonly recommended range of 10–20, suggesting an acceptable feature-to-event balance. Nevertheless, the ΔAUC of 0.065 between training (0.988) and validation (0.923) sets indicates that a degree of overfitting cannot be fully excluded, despite the use of LASSO regularization, cross-validated hyperparameter tuning, and stacking with a logistic-regression meta-learner.

Several limitations should be acknowledged. First, the retrospective, single-center design carries inherent risks of selection bias, and the homogeneous patient population may limit generalizability to settings with different demographics, clinical practices, or disease prevalence. Second, validation was internal (split-sample) only; prospective external validation across multiple centers and diverse populations remains essential before clinical deployment. Third, the full model incorporates VEGF, which is not routinely measured in many institutions; although the simplified 8-feature model partially mitigates this constraint, broader translation of the full model may be limited. Fourth, all enrolled patients underwent prophylactic central compartment dissection, so model performance in cohorts undergoing selective dissection based on clinical suspicion remains unknown. Fifth, the inclusion of a 1D-CNN on tabular clinical data is methodologically unconventional and should be regarded as exploratory rather than confirmatory. Finally, although LASSO regularization, cross-validated tuning, and ensemble averaging were used to mitigate overfitting, the ΔAUC of 0.065 between training and validation sets indicates that mild overfitting cannot be entirely excluded. The rapid advancement of AI techniques offers opportunities for further enhancement, including integration of multi-omics data and advanced imaging features center enrollment practice center (5, 30, 31).

In conclusion, our machine learning approach, combining stacking ensemble methods with LASSO feature selection and SHAP interpretability analysis, significantly improves CLNM prediction in PTC patients. The identification of inflammatory markers as key predictors provides new biological insights into lymph node metastasis mechanisms. The superior performance, robust generalizability, and clinical interpretability make this a promising tool for guiding surgical decision-making, potentially optimizing treatment strategies while reducing unnecessary surgical morbidity. This work represents an important step toward precision medicine in thyroid cancer care.

## Data Availability

The raw data supporting the conclusions of this article will be made available by the authors, without undue reservation.
