# Supplementary material for: Development and validation of an interpretable machine learning model for predicting central lymph node metastasis in papillary thyroid cancer
Source: Front Oncol. 2026 Jun 12;16:1839870. doi: 10.3389/fonc.2026.1839870 (PMC13303216; doi:10.3389/fonc.2026.1839870)
Supplement: Supplementary file 1 [file Table1.docx]

Table S1. Comparison of Clinical Characteristics Between CLNM and Non-CLNM Groups

| Variable | Non-CLNM (N=1181) | CLNM (N=516) | Z/t/χ² | *P* Value |
| --- | --- | --- | --- | --- |
| Demographics |  |  |  |  |
| Age (years) | 48 (38-56) | 43 (34-52) | 5.241 | <0.001 |
| Male sex, n (%) | 242 (20.5) | 128 (24.8) | 3.621 | 0.057 |
| Tumor Characteristics |  |  |  |  |
| Maximum tumor size (cm) | 0.7 (0.5-1.0) | 1.1 (0.8-1.6) | -12.358 | <0.001 |
| Multiple tumors, n (%) | 278 (23.5) | 199 (38.6) | 38.542 | <0.001 |
| Inflammatory Markers |  |  |  |  |
| NLR | 1.8 (1.4-2.2) | 2.0 (1.5-2.6) | -4.127 | <0.001 |
| PLR | 126.8 (103.5-160.2) | 142.5 (112.8-178.6) | -3.089 | 0.002 |
| LMR | 5.3 (4.3-6.5) | 4.7 (3.8-5.8) | 4.658 | <0.001 |
| MLR | 0.19 (0.15-0.24) | 0.21 (0.17-0.27) | -3.524 | <0.001 |
| SII | 385.2 (285.4-523.7) | 458.3 (338.6-635.2) | -4.892 | <0.001 |
| AISI | 0.6 (0.4-0.8) | 0.7 (0.5-1.0) | -2.147 | 0.032 |
| ELR | 0.05 (0.03-0.08) | 0.06 (0.03-0.09) | -1.524 | 0.128 |
| Hematological Parameters |  |  |  |  |
| WBC (×10⁹/L) | 5.4 (4.5-6.3) | 5.5 (4.6-6.5) | -1.236 | 0.216 |
| Neutrophil count (×10⁹/L) | 3.0 (2.5-3.8) | 3.3 (2.6-4.1) | -2.985 | 0.003 |
| Lymphocyte count (×10⁹/L) | 1.7 (1.4-2.1) | 1.7 (1.3-2.0) | 1.847 | 0.065 |
| Monocyte count (×10⁹/L) | 0.3 (0.3-0.4) | 0.4 (0.3-0.4) | -2.456 | 0.014 |
| Platelet count (×10⁹/L) | 222 (184-264) | 233 (192-274) | -2.108 | 0.035 |
| Hemoglobin (g/L) | 134 (125-144) | 133 (124-143) | 0.856 | 0.392 |
| Thyroid Function |  |  |  |  |
| TSH (mIU/L) | 2.0 (1.4-3.0) | 1.9 (1.3-2.8) | 1.724 | 0.085 |
| FT3 (pmol/L) | 5.0 (4.6-5.7) | 5.1 (4.6-5.8) | -0.982 | 0.326 |
| FT4 (pmol/L) | 15.9 (13.8-17.5) | 15.8 (13.5-17.6) | 0.425 | 0.671 |
| Thyroid Antibodies |  |  |  |  |
| Anti-TPO (IU/mL) | 12.4 (10.5-15.0) | 12.8 (10.8-15.8) | -1.447 | 0.148 |
| Anti-TG (IU/mL) | 16.4 (14.5-22.5) | 17.2 (15.0-24.5) | -1.892 | 0.058 |
| Hashimoto's thyroiditis, n (%) | 248 (21.0) | 111 (21.5) | 0.056 | 0.813 |
| Tumor Markers |  |  |  |  |
| Tg (ng/mL) | 13.8 (8.5-25.2) | 16.2 (9.8-30.5) | -2.547 | 0.011 |
| CEA (ng/mL) | 1.4 (0.9-1.9) | 1.4 (1.0-2.0) | -1.156 | 0.248 |
| VEGF (pg/mL) | 118.3 (118.3-118.3) | 118.3 (118.3-221.5) | -2.435 | 0.015 |
| Calcitonin (pg/mL) | 0.8 (0.5-2.9) | 0.7 (0.5-3.2) | -0.324 | 0.746 |
| Other Parameters |  |  |  |  |
| PTH (pg/mL) | 37.5 (27.6-47.2) | 37.8 (28.5-48.5) | -0.564 | 0.573 |
| 25(OH)D (ng/mL) | 15.4 (11.9-20.5) | 15.0 (11.5-19.8) | 1.235 | 0.217 |
| Vitamin D deficiency, n (%) | 871 (73.8) | 388 (75.2) | 0.385 | 0.535 |

Continuous variables presented as median (IQR); categorical variables as n (%). Abbreviations as in Table 1.
