# Supplementary material for: Development and validation of an interpretable machine learning model for predicting central lymph node metastasis in papillary thyroid cancer
Source: Front Oncol. 2026 Jun 12;16:1839870. doi: 10.3389/fonc.2026.1839870 (PMC13303216; doi:10.3389/fonc.2026.1839870)
Supplement: Supplementary file 2 [file Table2.docx]

Table S2. Subgroup Analysis of Stacking Ensemble Model Performance in Validation Set

| Subgroup | N | CLNM (%) | AUC (95% CI) | Accuracy | Sensitivity | Specificity | PPV | NPV | F1-Score |
| --- | --- | --- | --- | --- | --- | --- | --- | --- | --- |
| Overall Validation Set | 510 | 155 (30.4) | 0.923 (0.909-0.937) | 0.785 | 0.876 | 0.721 | 0.678 | 0.938 | 0.764 |
|  |  |  |  |  |  |  |  |  |  |
| Tumor Size Stratification | | |  |  |  |  |  |  |  |
| ≤1.0 cm | 327 | 79 (24.2) | 0.901 (0.878-0.924) | 0.768 | 0.848 | 0.734 | 0.587 | 0.918 | 0.695 |
| >1.0 cm | 183 | 76 (41.5) | 0.935 (0.915-0.955) | 0.813 | 0.895 | 0.720 | 0.776 | 0.881 | 0.831 |
| *P* value |  |  | 0.082 |  |  |  |  |  |  |
|  |  |  |  |  |  |  |  |  |  |
| Hashimoto's Thyroiditis Status | | |  |  |  |  |  |  |  |
| Present | 105 | 29 (27.6) | 0.918 (0.892-0.944) | 0.781 | 0.862 | 0.750 | 0.610 | 0.927 | 0.714 |
| Absent | 405 | 126 (31.1) | 0.925 (0.908-0.942) | 0.786 | 0.881 | 0.715 | 0.695 | 0.890 | 0.777 |
| *P* value |  |  | 0.697 |  |  |  |  |  |  |
|  |  |  |  |  |  |  |  |  |  |
| Age Stratification |  |  |  |  |  |  |  |  |  |
| ≤45 years | 257 | 86 (33.5) | 0.931 (0.912-0.950) | 0.799 | 0.895 | 0.743 | 0.699 | 0.921 | 0.785 |
| >45 years | 253 | 69 (27.3) | 0.916 (0.895-0.937) | 0.770 | 0.855 | 0.712 | 0.614 | 0.901 | 0.713 |
| *P* value |  |  | 0.328 |  |  |  |  |  |  |
